# Despite mutation acquisition in hematopoietic stem cells, JMML-propagating cells are not always restricted to this compartment

**DOI:** 10.1038/s41375-019-0662-y

**Published:** 2019-11-27

**Authors:** Aurélie Caye, Kevin Rouault-Pierre, Marion Strullu, Elodie Lainey, Ander Abarrategi, Odile Fenneteau, Chloé Arfeuille, Jennifer Osman, Bruno Cassinat, Sabrina Pereira, Fernando Anjos-Afonso, Erin Currie, Linda Ariza-McNaughton, Vincent Barlogis, Jean-Hugues Dalle, André Baruchel, Christine Chomienne, Hélène Cavé, Dominique Bonnet

**Affiliations:** 1 INSERM UMR_S1131, Institut de Recherche Saint-Louis, Université de Paris, Paris, France; 2 Département de Génétique, Hôpital Robert Debré, Assistance Publique des Hôpitaux de Paris (AP-HP), Paris, France; 3 Francis Crick Institute, London, UK; 4 Service d’Hématologie Biologique, Hôpital Robert Debré, Assistance Publique des Hôpitaux de Paris (AP-HP), Paris, France; 5 Service de Biologie Cellulaire, Hôpital Saint Louis, Assistance Publique des Hôpitaux de Paris (AP-HP), Paris, France; 6 Service d’Hématologie Pédiatrique, Hôpital de la Timone, Assistance Publique des Hôpitaux de Marseille (AP-HM), Marseille, France; 7 Service d’Hématologie pédiatrique, Hôpital Robert Debré, Assistance Publique des Hôpitaux de Paris (AP-HP), Paris, France; 8 Present Address: Barts Cancer Institute, Centre for Haemato-Oncology, Queen Mary University of London, London, UK

**Keywords:** Cancer stem cells, Myelodysplastic syndrome

## Abstract

Juvenile myelomonocytic leukemia (JMML) is a rare aggressive myelodysplastic/myeloproliferative neoplasm of early childhood, initiated by RAS-activating mutations. Genomic analyses have recently described JMML mutational landscape; however, the nature of JMML-propagating cells (JMML-PCs) and the clonal architecture of the disease remained until now elusive. Combining genomic (exome, RNA-seq), Colony forming assay and xenograft studies, we detect the presence of JMML-PCs that faithfully reproduce JMML features including the complex/nonlinear organization of dominant/minor clones, both at diagnosis and relapse. Further integrated analysis also reveals that although the mutations are acquired in hematopoietic stem cells, JMML-PCs are not always restricted to this compartment, highlighting the heterogeneity of the disease during the initiation steps. We show that the hematopoietic stem/progenitor cell phenotype is globally maintained in JMML despite overexpression of CD90/THY-1 in a subset of patients. This study shed new lights into the ontogeny of JMML, and the identity of JMML-PCs, and provides robust models to monitor the disease and test novel therapeutic approaches.

## Introduction

Juvenile myelomonocytic leukemia (JMML) is a rare and aggressive childhood myelodysplastic/myeloproliferative neoplasm (MDS/MPN) thought to be initiated by the activation of the RAS signal transduction pathway due to germline or somatic mutations in genes encoding RAS (*NRAS*, *KRAS*) or RAS-pathway regulators (*PTPN11* encoding the SHP2 cytoplasmic phosphatase, and less frequently *NF1* or *CBL)* [1, 2]. A hallmark of JMML is a hypersensitivity to granulocyte macrophage-colony stimulating factor (GM-CSF), which leads to enhanced *in vitro* proliferation of monocyte-macrophage colonies in the absence of exogenous colony stimulating factor [3, 4]. In patients, this excessive proliferation of monocytes and granulocytes leads to hepatosplenomegaly, lymphadenopathy, skin rash, and respiratory failure. JMML is usually rapidly fatal due to multiorgan failure or progression towards acute myeloid leukemia (AML) unless allogeneic hematopoietic stem cell transplantation (HSCT) is performed [5]. However, there is a significant risk of post-HSCT recurrence, and overall survival only reaches 50–60% [6].

Previous studies [7–9] based on whole exome sequencing (WES) have uncovered additional genetic abnormalities in about 65% of sporadic JMML cases and demonstrated the association between JMML outcome and mutational profile. Indeed, the presence of more than one RAS-activating mutation (RAS double mutants) distinguishes very aggressive JMML with an increased risk of AML progression [7–9].

If the genomic landscape of JMML is relatively well defined today, very little is known about the origin of JMML, the JMML-propagating cell (JMML-PC) or the clonal evolution in JMML, those being, all crucial steps to improve the management of children with JMML.

Although this syndrome is predominantly characterized by granulo-monocytic lineage involvement, JMML also shows evidence of multilineage involvement [10]. Thrombocytopenia is a common feature, high fetal hemoglobin levels are found in about 60% of patients, reflecting a contribution of the erythroid lineage [11], and cases of JMML evolving into a B lymphoid blast crisis have been reported [12]. JMML is thus considered a disease of the hematopoietic stem/progenitor cell (HSPC) compartment. However, the putative leukemia-propagating cells have not yet been characterized, and evidence of clonal heterogeneity, with lymphoid lineage cells harboring RAS-activating mutations in some patients but not in others, suggests that the cell type affected by the initiating mutation may vary between patients [2].

Patient-derived xenografts (PDX) provide an invaluable tool to functionally assess tumor samples for leukemia-propagating cell potential. However, limited data are available concerning the capacity of primary JMML PDX to engraft in immunodeficient mice, and whether addition of exogenous human GM-CSF is required for JMML to engraft is not clear [13–16]. Moreover, no study to date has established the robustness of the JMML xenotransplantation model with regard to the reproducibility of the disease, clonal composition, and clonal evolution in mice. Importantly, the identity of the cells capable of propagating JMML in vivo at diagnosis and at relapse has yet to be defined. Lastly, the cell type in which oncogenic mutations arise and the dynamics of clonal expansion during hematopoietic differentiation remains to be determined.

In this paper, we aimed to address these questions by using a combination of whole-exome and targeted deep next-generation sequencing, single-cell colony analysis, and cell sorting as well as xenotransplantation experiments with 17 JMML samples obtained from 15 patients.

## Material and methods

### Patient samples

The cohort included 36 patients with JMML (19 males, 17 females) aged 3 months to 13 years (median 2.2 years) (Supplementary Table S1). All patients fulfilled the WHO consensus JMML criteria [17]. Most patients had sporadic JMML with mutations in *PTPN11* (PTPN11-JMML; *n* = 13), *NRAS* (NRAS-JMML; *n* = 14), *KRAS* (KRAS-JMML; *n* = 5), or other genes (*n* = 3). An additional patient (#123) had a germline *PTPN11* p.N308T mutation, in line with Noonan syndrome features.

JMML patient samples (BM, *n* = 36; peripheral blood *n* = 2) were collected in a diagnostic setting. All children’s samples were obtained after parents had given their written informed consent. This study was approved by the institutional review board of the French Institute of Health and Medical Research (INSERM) (IORG0003254) in accordance with the Helsinki declaration.

Clinical data and genetic profiles obtained from 15 of these JMML patients that were studied using xenotransplantation models are shown Table 1. Pulmonary involvement was determined as clinical signs of respiratory distress ± documented leukemia infiltration. Blast crisis was defined as the presence of ≥20% blasts assessed by cytomorphological examination of the BM. A second RAS-activating mutation was observed in 6/15 patients including duplication of the oncogenic mutation due to acquired uniparental disomy (aUPD) in two cases.

**Table 1:**
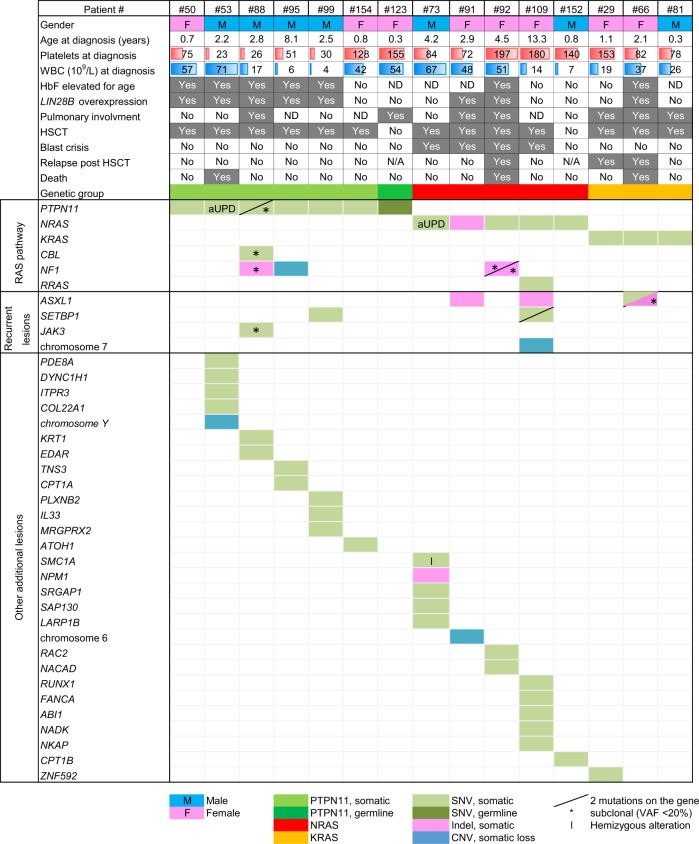
Clinical data and detailed genetic profiles obtained by combining karyotype, genome-wide DNA array analysis, whole exome (WES) and targeted next-generation sequencing for the 15 JMML patients who were studied using xenotransplantation in mouse

Each column highlights the condensed data for a single patient at diagnosis

*WBC* whole blood cell count, *HbF* fetal hemoglobin, *HSCT* hematopoietic stem cell transplantation, *ND* not done, *N/A* not applicable, *SNV* single-nucleotide variation, *CNV* copy number variation, *aUPD* acquired uniparental disomy, *VAF* variant allele frequency

Dash: two mutations on the same gene

*Subclonal alteration (VAF < 20%); I: Hemizygous alteration

BM of healthy age-matched children (*n* = 19) was obtained from intrafamilial BM transplantation donors. The collection and use of these samples were approved by the Institutional Review Board of “Hôpitaux Universitaires Paris Nord Val-de-Seine,” Paris 7 University, AP-HP), (IRB: 00006477), in accordance with the Helsinki declaration.

### Animals

All animal experiments were performed at the Francis Crick Institute in accordance with UK Home Office and CRICK guidelines and were undertaken under the Home Office project license PLL 70/8904. NOD/SCID/IL2rγ^−/−^ (NSG) mice and NOD/SCID/IL2rγ^−/−^/IL-3/GM/SF (NSG-S) mice were originally a kind gift of Dr Leonard Shultz (The Jackson Laboratory) and since then have been bred at the Francis Crick Institute Biological Resource facility. These mice were genotypes on arrival and on regular basis thereafter using the genotypic protocols provided by the Jackson laboratory.

### Xenotransplantation

Prior to transplantation, NOD/SCID/IL2rγ^−/−^ (NSG) mice and NOD/SCID/IL2rγ^−/−^/IL-3/GM/SF (NSG-S) mice (aged 8–12 weeks old), sex matched/patient (female or male) received a sublethal dose of radiation (350–375 cGy) from a cesium-137 source. Direct intra-BM injection was performed in the tibia with 15 × 10^3^ BM CD34^+^ cells from patients and healthy donors. Number of mice injected per samples varied between samples and were dependent on the primary cells available (from two to six mice). Neither exclusion criteria nor randomization were performed. The investigator was not blind to the group. Engraftment was assessed at sacrifice (6–12 weeks) and the BM cells (pooled femurs, tibias, and 1 pelvis) were immuno-phenotyped for the presence of mCD45, hCD45, hCD33, hCD19, and hCD3 (BD Biosciences, Oxford, UK) cell populations. Live cells were stained and sorted on hCD45 phenotype using FACS Aria SORP (BD Biosciences, Oxford, UK) or sorted by magnet isolation using EasySep™ Mouse/Human chimera isolation kit (cat no. 19849, Stem cell technologies, Inc, Vancouver, Canada). Isolated CD45^+^ cells were washed in PBS and plated into methylcellulose as mentioned below and/or pelleted in order to later perform genomic analysis or used for secondary transplantation experiment. In addition, one pelvis, one lobe of lung and the spleen were fixed for immunochemistry/immunofluorescence and 10^5^ cells from harvested BM were cytospinned, and then fixed in methanol on a slide for later analysis using May–Grunwald–Giemsa staining. For secondary transplantation, between 1 and 3.7 × 10^6^, human CD45^+^ cells were injected into sublethally irradiated recipient mice (NSG or NSG-S).

### Depositing dataset

Whole genome sequencing data were available via ArrayExpress database: http://www.ebi.ac.uk/arrayexpress, Accession number E-MTAB-6461 (patients) and E-MTAB-6467 (xenograft samples). For SNP/CGH array: Accession numbers E-MTAB-3729 (SNP array) and E-MTAB-6468 (CGH + SNP array).

### Statistics

Differences between groups were tested using the Mann–Whitney test (two groups) or by using multivariable one-way ANOVA unpaired analysis corrected for multiple comparisons (multiple groups). All analyses were performed with Prism software. version 6.0 (GraphPad, La Jolla, CA, USA). Statistical significance was defined as *p* value < 0.05. Statistical value is provided in each figure.

Supplementary material and methods section is available in supplementary information including reagents and resources used in this paper (see Table S8).

## Results

### JMML bone marrow cells maintain HSPC phenotype

**Fig. 1:**
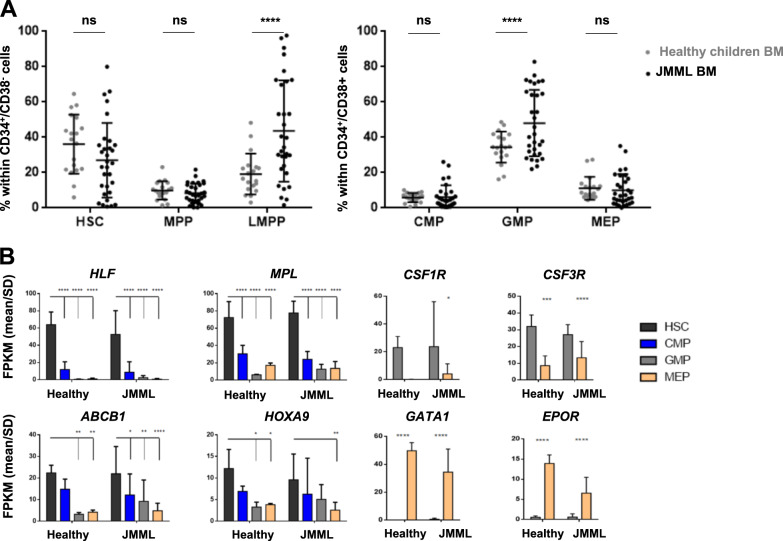
Despite heterogenous distribution, phenotypically and molecularly defined stem/progenitor cell fractions are maintained in JMML. **a** Distribution of phenotypically defined HSC, MPP, and LMPP within the CD34^+^CD38^−^ population (left panel), and of CMP, GMP, and MEP within the CD34^+^CD38^+^ population (right panel) in BM of patients with JMML (*n* = 31) compared with healthy children (*n* = 19). **b** Transcriptional validation of phenotypically defined JMML stem/progenitor cell fractions (*n* = 14) compared with their normal counterparts sorted from healthy children BM (*n* = 4). RNAseq results are expressed as mean FPKM scores (±SD) for gene transcripts that are characteristic of the normal counterpart of phenotypically defined stem (*HLF, MPL, ABCB1,* and *HOXA9*) and progenitor cell (*CSF1R, CSF3R, EPO,* and *GATA1*) fractions. See Supplementary Fig. S2 for gating strategy and RNAseq results for additional genes. Anova multiple comparison, ****p < 0.0001; ***p < 0.001; **p < 0.01; *p < 0.05; ns not significant, BM bone marrow, FPKM fragments per kilobase million, SD standard deviation

JMML BM samples (*n* = 31) (Supplementary Table S1) were compared with BM from age-matched healthy children (*n* = 19). Hematopoietic stem cells (HSCs), multipotent progenitors (MPPs), lymphoid-primed multipotent progenitors (LMPPs), common myeloid progenitors (CMPs), granulocyte-macrophage progenitors (GMPs), and megakaryocyte-erythroid progenitors (MEPs) were identified using multiparametric FACS analyses based on previously defined markers [18] (Fig. 1 and Supplementary Fig. S1A). JMML BM displayed a significant expansion of the LMPP and GMP compartments (Fig. 1a and Supplementary Fig. S1B). This expansion was mostly accounted for by *PTPN11*-mutated JMML, which also showed a larger percentage of myeloid progenitors compared with healthy BM within the CD34^+^CD38^+^ compartment (Supplementary Fig. S1C), consistent with the major granulo-monocytic expansion observed in patients with this subtype of JMML. However, despite the expected LMPP/GMP trend, all hematopoietic compartments (phenotypically and molecularly defined) were present in most JMML samples, as in their healthy counterparts (Fig. 1b and Supplementary Fig. S1D), thus showing that the HSPC phenotype is globally maintained in bone marrow of JMML patients.

**Fig. 2:**
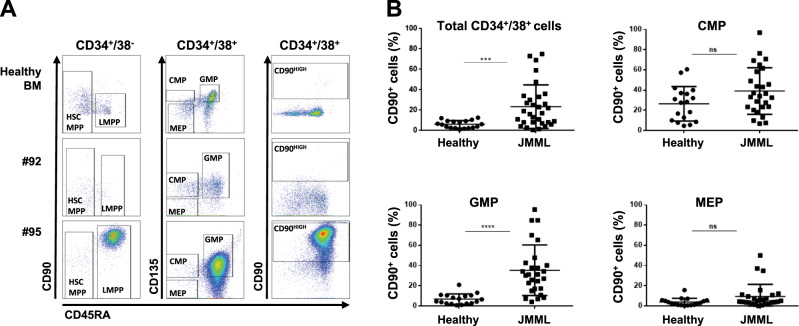
CD90 is overexpressed in a subset of JMML. **a** Representative flow cytometry plots of a healthy child BM, and patients #92 (*NRAS*-JMML) and #95 (*PTPN11*-JMML) with respectively normal and over/ectopic expression of CD90/Thy1 across the different hematopoietic compartments. The first column shows CD90 vs CD45RA within the CD34^+^CD38^−^ fraction, the middle column shows CD135 vs CD45RA and last column shows CD90 vs CD45RA within the CD34^+^CD38^+^ fraction. Gating of cellular HSPC fractions is indicated. **b** Percentage of CD90/Thy1 expressing cells measured by flow cytometry in JMML compared with healthy children BM within the total CD34^+^CD38^+^ fraction, and across the different hematopoietic progenitor compartments CMP, GMP, MEP (see also Supplementary Table S2). Anova multiple comparison, ****p < 0.0001; ***p < 0.001; ns not significant

This analysis also revealed an aberrant upregulation of CD90/Thy1 expression within the CD34^+^CD38^−^ fraction in a subset of JMML cases, that persisted in the CD34^+^/38^+^ progenitor fraction (Fig. 2a, b and Supplementary Table S2), and could be tracked down to the monocyte population in some patients (not shown). Within CD34^+^/38^+^ progenitors, aberrant CD90 expression was mostly found in the GMP-like population, with 15/27 (55%) of JMML cases showing ectopic CD90 expression in more than 30% of the GMP cells. This was confirmed as a transcriptional deregulation by RNAseq analysis (data not shown).

### JMML is recapitulated in both NSG and NSG-S mice

In order to functionally assess the leukemia-propagating cell potential, xenotransplantation of cells from 15 genetically defined JMML was performed (Table 1 and Supplementary Table S1). Consistent with the specific hypersensitivity of JMML myeloid progenitors to GM-CSF [3], the treatment of immunodeficient mice with human GM-CSF has been shown to favor the engraftment of JMML cells [15]. Xenotransplantation of cells from ten JMML samples at diagnosis and two JMML samples at relapse was thus performed in parallel in two immunocompromised mouse strains NSG mice and NSG-S mice (also called NSG-SGM3), which is humanized to express the cytokines GM-CSF, SCF and IL-3 (Supplementary Table S1). Two more JMML samples were injected in NSG mice only, and one in NSG-S mice only.

In compliance with UK Home Office guidelines, we first investigated, using samples from a small cohort of four patients (see Supplementary Fig. S2A, B), how long mice of each strain could be maintained in a healthy state post transplantation. The overall sickness curve showed that NSG-S mice had to be sacrificed after ~6 weeks, compared with 12 weeks for NSG mice (Supplementary Fig. S2A). At 6 weeks, the level of engraftment in NSG mice was below the detection threshold (<0.1%) for two out of the four xenografts tested (Supplementary Fig. S2B). Accordingly, we decided to sacrifice the NSG-S mice at 6 weeks and the NSG mice at 12 weeks (or earlier if signs of sickness were observed in any of the littermates).

**Fig. 3:**
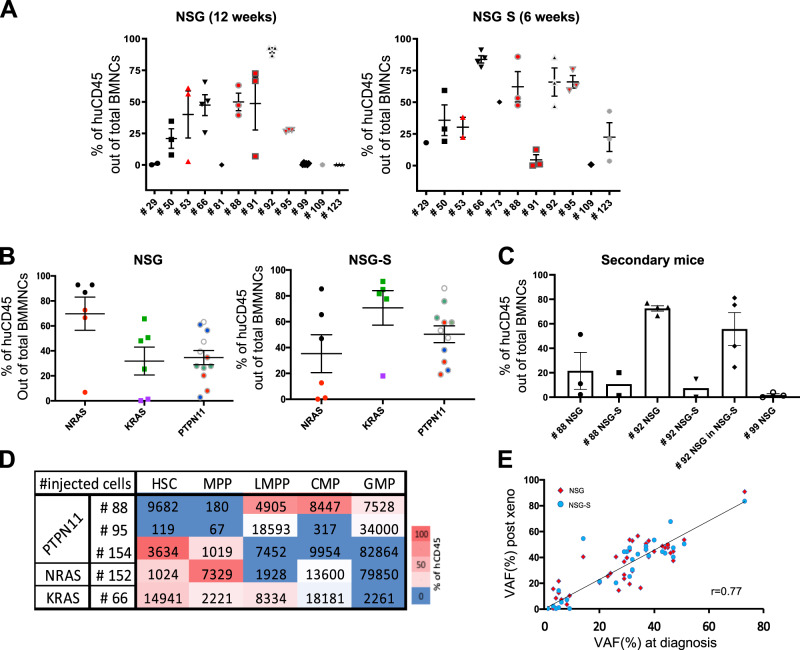
PDX models accurately capture the features and clonal diversity of the disease and allow to characterize the JMML-PC. **a** Percentage of human CD45 out of total BMNCs present in the mouse BM at termination, in NSG (*n* = 12 patients, *n* = 35 mice), and NSG-S (*n* = 11 patients, *n* = 27 mice). In each panel, red symbols indicate JMML that are CD90^high^ (#53, #88, #91, and #95). **b** The levels of human engraftment are displayed per mutated gene between the two mice models. Matching shapes and colors represent the same patient between the two models. **c** The level of human CD45 engraftment out of total nucleated cells in secondary recipients (10 NSG, 8 NSG-S) for three patients (#88, #92, and #99). Samples harvested from primary NSG mice were injected into NSG secondary recipient and/or primary NSG-S into secondary NSG-S. For patient #92, cells harvested from the NSG primary mouse were injected into either NSG or NSG-S (see also Supplementary Table S3). **d** Heat map representation of the level of engraftment obtained after injection of the different JMML hematopoietic fractions (HSC, MPP, LMPP, CMP, GMP) of five patients with JMML (#88, #95, #154, #152, and #66). The number of cells injected per patient and per fraction is displayed for each fraction. **e** Correlation of variant allele frequencies (VAF) obtained from the cells post xenotransplant compared with the native JMML cells from the patient at diagnostic. Each dot representing one mutation (red dots: NSG; blue dots: NSG-S). See also Supplementary Fig. S3

In NSG, 4 samples out of 12 tested were below our threshold (<0.1%) of detection compared with only 1 out of 11 in NSG-S. Only one patient sample (#109) did not engraft in either mouse models. Two samples that were not engrafted in NSG at 12 weeks, engrafted in NSG-S mice at 6 weeks. However, in all other cases, engraftment was detected in both models (Fig. 3a). The kinetics and level of engraftment observed in NSG and NSG-S, varied between patients and according to the initiating JMML mutations (Fig. 3a). Interestingly, despite the maintenance of the CD90^high^ expression in the cells out of the mice (data not shown), we could not evidence a higher engraftment capacity of the CD90^high^ JMML samples tested (see Fig. 3a, patients 53, 88, 91, and 95).

At sacrifice, mouse BM showed characteristic JMML cytomorphological features, with similar blasts and myelomonocytic cell counts to the native sample (Supplementary Fig. S2D). Engrafted mice also had splenomegaly, which was more prominent in NSG-S mice (Supplementary Fig. S2E). Consistent with the pulmonary involvement frequently observed in JMML patients, immunofluorescence labeling of murine lungs additionally revealed human CD45^+^ cell infiltration after transplantation with some of the JMML samples (Supplementary Fig. S2F).

Thus, both mouse models recapitulate unique clinical features of the JMML pathology, with a general faster engraftment in NSG-S mice highlighting the hypersensitivity of JMML cells to GM-CSF.

### JMML xenografts identify multiple JMML-PCs that may reside in non-HSC fractions

To further confirm the presence of JMML-PCs in the two models, we tested their self-renewal potential by performing secondary transplantation of three JMML transplants (#88, #92, and #99), into the matching mouse strain. In order to understand whether human cytokines present in NSG-S would increase engraftment in secondary mice, one JMML sample (#92), first engrafted in an NSG mouse, was cross-transplanted into an NSG-S mouse. In all cases, successful secondary engraftment was obtained at 12 weeks (Fig. 3c and Supplementary Table S3).

Contrary to what was observed with primary transplants, engraftment was slightly decreased following secondary transplantation in NSG-S compared with NSG mice despite the longer time-lapse in NSG compared with NSG-S mice (12 + 12 weeks for NSG versus 6 + 12 weeks for NSG-S) suggesting that the self-renewing JMML-PCs are less sensitive to human cytokines.

Overall, our data evidence the presence of self-renewing JMML-PCs and confirm the robustness of both mouse models in promoting engraftment and maintaining JMML-PCs, although NSG mice present some advantages in terms of survival times.

Having proven the presence of self-renewing JMML-PCs in JMML samples, we next investigated the nature of these cells. HSC, MPP, LMPP, CMP, and GMP subfractions were sorted from five JMML patients and injected separately into NSG mice. NSG mice were preferred to NSG-S mice since we wanted to reveal JMML long-term propagating cells. In most patients from whom JMML cells engrafted, xenografts confirmed the presence of JMML-PCs in the more immature HSC/MPP compartment. Intriguingly, more committed cells, such as LMPP/CMP/GMP, were also able to propagate the disease, demonstrating the JMML-PC capacity of the mature compartment (Fig. 3d). From these data, it is clear that JMML-PCs are not restricted to the HSC fractions, and that in the same patients, more than one subtype of JMML-PCs could be found.

### JMML xenografts maintain the JMML mutational landscape in both mouse models

The mutational landscape of JMML xenografts, investigated by WES, was compared with the patient sample pre transplantation (Supplementary Tables S4 and S5). The allelic frequency of the identified pathogenic variants (VAF) was further determined using targeted high-throughput sequencing (Supplementary Table S5).

With an overall correlation rate of 0.77 for VAF measured before and after transplantation in NSG and NSG-S mice (Fig. 3e), our data show that xenotransplantation preserved the overall mutational profile of the native JMML samples both in terms of clonal diversity and distribution. Only a few native variants with a low allelic burden remained undetected in mice (Supplementary Fig. S3), likely reflecting a stochastic process related to the low number of injected cells rather than to a counter-selection in mice.

No difference in the mutational landscape was observed between NSG and NSG-S mice (Fig. 3e, Supplementary Fig. S3, and Table S5), indicating that the human cytokines present in the latter, despite stimulating JMML cell growth, did not impact their clonal distribution. No novel variant was detected in xenotransplanted mice that was not already present in the native sample.

We also confirmed that the cells present in the secondary transplanted mice had a similar clonal composition to those present in the original sample (Supplementary Table S5).

### JMML subclonal mutation architecture reveals early clonal dominance

**Fig. 4:**
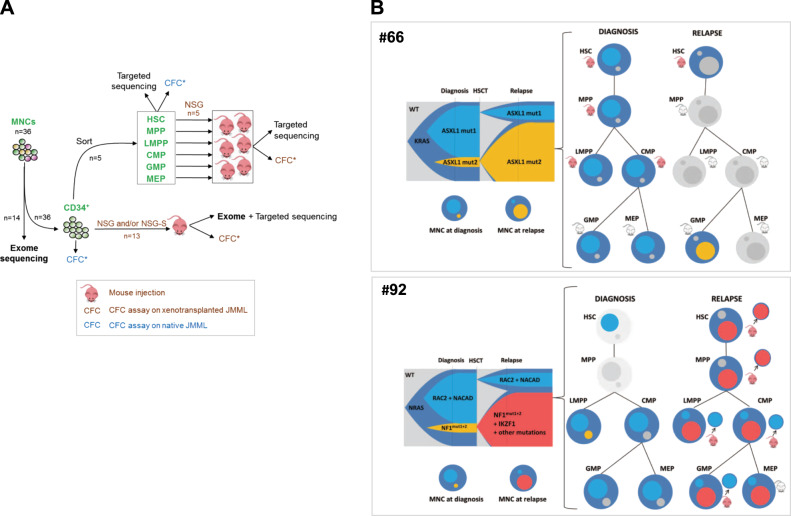
Clonal dynamics with time and across hematopoietic differentiation shows early clonal dominance. **a** Schematic of experimental procedure followed in order to delineate the origin and clonal architecture of JMML. All CFCs obtained from these experiments on naïve JMML (blue icons) or xenotransplanted JMML (brown icons) were tested for known patient mutations by targeted sequencing. See also Fig. S4. **b** Clonal architecture of two JMML samples (#66 and #92), as determined by combining whole exome sequencing, deep targeted sequencing, and single-cell derived colony sequencing before and after xenotransplantation. For each patient, a fish plot (left) represents clonal evolution between diagnosis and relapse. In the absence of preleukemia sample allowing to specify the kinetics of clonal emergence, subclones were represented by default as appearing simultaneously. Mutations found in each subclone are indicated (see also Supplementary Table S6). The clonal composition in total JMML mononucleated cells (MNCs) at diagnosis or relapse is also represented in circles. The larger circle represents the founding clone. Smaller circles inside represent subclones of various size and matching colors with the fish plot. Clonal composition and engraftment capacities across hematopoietic differentiation are represented on the right panels. Mutations identified in MNC were screened in sorted fractions before and after xenotransplantation using Sanger sequencing. Mouse icons tag fractions that were injected in NSG and/or NSG-S mice. Red mouse icons indicate successful engraftment whereas gray icons indicate engraftment failure. Patient (#66) KRAS-JMML showing branched evolution with independent acquisition of additional mutations targeting *ASXL1*. The dominant clone at diagnosis or at relapse was also dominant in corresponding xenografts. Patient (#92) NRAS-JMML showing branched evolution with independent acquisition of additional mutations targeting *either NF1* or *RAC2*. At relapse, exome sequencing performed on the MNC evidenced the gain of an *IKZF1* mutation within the clone that became dominant at relapse. Sorted relapse HSC, MPP, and LMPP engrafted in NSG mice and targeted sequencing of individual picked CFC obtained from these mice demonstrated the presence of the dominant relapse clone in HSC and MPP whereas cells retrieved from the LMPP, CMP, and GMP engrafted mice only harbored the mutations of the clone that was dominant at diagnosis but minor at relapse

To gain further insight into the clonal structure of JMML, we identified the combinatorial patterns of “driver mutations” in individual clones using VAF determined by deep sequencing of JMML, in combination with the sequencing of single-cell derived colonies obtained from clonogenic (colony forming cell, CFC) assays, performed before and after engraftment in mice (Fig. 4a).

Two groups of JMML could be delineated according to their clonal architecture. A majority of patients (12/15) presented at diagnosis with either a single (3/12), or several mutations co-existing in a unique JMML clone (9/12), as demonstrated by CFC analysis and/or postulated from elevated VAF (>30%) for all variants (data not shown). This was consistent with a linear pattern of mutation acquisition, although in most cases, the order of acquisition of mutations could not be determined since they were all present in each clone that could be studied (see example of patient 95, Supplementary Fig. S4).

In the remaining patients (3/15), JMML showed nonlinear progression with several subclones co-existing at diagnosis (Fig. 4b, patients 66 and 92 and Supplementary Fig. S4, patient 88). Interestingly, NSG and NSG-S models faithfully captured this clonal heterogeneity in all patients, with all subclones identified in the xenotransplanted cells (see CFC assays from NSG or NSG-S mice, Supplementary Table S6).

We then investigated the dynamics of JMML clonal architecture during myeloid differentiation by comparing clonal frequencies in sorted HSC, MPP, LMPP, CMP, GMP, and MEP cells from five patients with both types of clonal architecture, before and after engraftment in mice (Supplementary Table S1 and Fig. 4a). Whether the JMML showed linear (*n* = 2) or branched (*n* = 3) clonal evolution, targeted sequencing of sorted fractions evidenced the presence of all mutations in the HSC or CD34^+^CD38^−^ compartment, as well as in more differentiated fractions, with frequencies mirroring those found in the bulk of mononucleated cells (Supplementary Table S7). The same was observed when analyzing the global mutational landscape present in mice injected with different JMML-PC-containing cell fractions from the same patient. Not only were all mutations (initiating as well as additional) found in all cell fractions that could be tested, but the balance between subclones also seemed to be preserved in most fractions at diagnosis (Supplementary Table S7).

These data are consistent with an early dominance of the leukemic clone during hematopoiesis and suggest that not only the initiating mutation but also additional ones contribute to this early clonal dominance, with a stable clonal distribution that remains unaffected during hematopoietic differentiation.

Two (#66 and #92) of the three patients with a branched clonal architecture at diagnosis subsequently relapsed. Patient 66’s JMML displayed branched evolution from a *KRAS*-mutated founder clone. A clonal outgrowth was observed at relapse, with a minor clone at diagnosis becoming the dominant one (Fig. 4b and Supplementary Table S6). Whatever the fraction injected in the mice (total CD34^+^ cells or sorted HSC, MPP, and LMPP), direct sequencing of human engrafted CD45^+^ cells and CFC analysis out of the mice revealed that the clonal architecture of the native JMML sample (whether at diagnosis or relapse) was maintained in mice (Fig. 4b and Supplementary Table S6).

Patient 92’s JMML also displayed branched evolution from an *NRAS*-mutated founder clone with a major subclone having acquired mutations in *RAC2* and *NACAD* and a minor subclone harboring two distinct mutations in *NF1* (Fig. 4b and Supplementary Table S6). At relapse, this latter subclone gained a mutation in *IKZF1* and became dominant (Fig. 4b and Supplementary Table S6). Here again, when total CD34^+^ cells were injected, the clonal architecture of the native JMML sample was maintained in mice regardless of the stage at which the JMML was sampled. However, when HSPC fractions from Patient 92’s relapse sample were injected in mice, only human CD45^+^ cells obtained from HSC- and MPP-injected mice corresponded to the relapse clone. Indeed, analysis of cells retrieved from the LMPP- and progenitor fraction-engrafted mice only evidenced the clone dominant at diagnosis but not the new clone that arose at relapse (Fig. 4b and Supplementary Table S6). Thus, in this patient, JMML-PCs, depending on maturational stage, had two different mutation signatures, the HSCs and MPPs propagated the relapse clone signature only, whereas the JMML-PCs with an LMPP/progenitor phenotype harbored the clone signature at diagnosis. This suggests that the clonal evolution observed at relapse is associated with a loss of the engraftment property of the most mature hematopoietic fractions.

## Discussion

The nature and exact stage of hematopoietic differentiation of the cell of origin in JMML remains elusive. Better knowledge of the origin of the JMML-PC as well as its clonal organization and integration within the hematopoietic differentiation hierarchy could be instrumental in providing insight into the pathogenesis of this severe but still poorly understood leukemia. Xenotransplantation in immunodeficient mice combined with CFC assays, cell sorting and genomics is a powerful tool for leukemia-initiating cell characterization. We therefore decided to evaluate the engraftment capacity of JMML in immunodeficient mice by comparing transgenic NSG and NSG-S mice.

In vivo xenotransplantation into NSG and/or NSG-S mice of samples from 15 JMML cases covering the most frequent genetic subgroups (i.e., PTPN11, NRAS, and KRAS) confirmed the capacity of most JMML samples to engraft in immunodeficient mice [14, 16]. Injection of isolated HSPC fractions in immunocompromised mice revealed that despite the HSC origin of the whole mutational landscape, JMML-PCs were not restricted to this fraction but could be present in MPPs, LMPPs and even in progenitor cells such as CMPs/GMPs. Also, and quite surprisingly, in samples from the same patient, more than one subtype of JMML-PCs could be detected, highlighting the challenge of eradicating these cells in JMML patients. Importantly, these findings are not the consequence of phenotypic unfaithfulness since we show that the HSPC hierarchy is preserved in JMML, similar to what has previously been shown in other types of MDS/MPN [19].

Our data demonstrate the robustness of the JMML xenotransplantation model and show that both mouse models not only reproduce major JMML clinical features, but also respect the native clonal architecture in all three major genetic groups of sporadic JMML. Such findings are in sharp contrast with what has been reported for AML [19, 20] or T-cell acute lymphoblastic leukemia [21], and suggests that minor subclones that may have remained undetected in the native sample either do not exist in JMML or are not selected differently in mice than in the patient. Strikingly, the mouse models did not anticipate clonal evolution observed at JMML relapse. However, the clonal shift at relapse might well be stochastic, due to the drastic clonal reduction induced by BM transplantation.

Interestingly, whatever the initiating genetic lesion, GM-CSF expressed by NSG-S mice leads to a rapid myeloid expansion, contributing to the aggressive nature of the malignancy, but does not have a significant long-term effect on self-renewing JMML-PCs. Importantly, NSG-S mice also do not seem to induce a bias in clonality compared with NSG, contrary to what might have been expected if human GM-CSF preferentially “overstimulated” specific subclones. This highlights that, although GM-CSF hypersensitivity is a well-known hallmark of JMML, it is more likely a consequence favoring the granulo-monocytic expansion than a causative abnormality in JMML-PCs. This is consistent with the lack of activating GM-CSF mutation in JMML [7–9] and suggests that a therapeutic strategy antagonizing GM-CSF [13] might efficiently limit JMML proliferation but probably not eradicate the disease.

Integration of the hematopoietic cellular hierarchy with clonal evolution by analyzing isolated stem cell and progenitor fractions, showed that the complete JMML mutational profile is already present with a high allelic burden in the HSC/LMPP compartment, consistent with early clonal dominance. In chronic myelomonocytic leukemia, an MPN resembling JMML but occurring in adults [22], such an early clonal dominance was also observed but the genetic diversity captured by HSCs was shown to be lost in downstream progenitor subsets due to systematic out-competition of a dominant subclone in the GMP compartment [23]. In contrast, we show that in JMML, the mutational landscape as well as the clonal equilibrium are stable throughout the myeloid differentiation, even though the LMPP and GMP compartments were amplified in a subset of patients. This suggests that clonal selection occurred within the HSC/MPP compartment very early in the oncogenic process and was not affected by hematopoietic differentiation or expansion, highlighting the stem cell origin of the JMML despite the complexity of JMML-PCs.

Finally, this study highlighted an aberrant overexpression of CD90 in a subset of patients. CD90/Thy-1 is a cell surface glycoprotein involved in cell adhesion and cell communication in numerous cell types. Although CD90 is considered a major marker of HSC pluripotency and a surrogate marker for HSCs, we could not evidence a higher engraftment capacity in these CD90^high^ JMML samples. Further work will be needed to determine the potential biological or clinical relevance of the upregulation of this marker in some patients.

In conclusion, our data provides new evidence of JMML heterogeneity, unveiling a complex clonal architecture and different JMML-PCs. This heterogeneity could not be explained by the pattern of genetic alterations nor by the initiating cell in which mutations arise. In addition, we show here that, by faithfully recapitulating both the key clinical features and unbiased clonal architecture of the disease, xenotransplanted mice could provide an invaluable model for further research and testing of new candidate treatments, which are urgently needed in this still mostly incurable disease.

## Supplementary information

Supplementary data
