# Correction: Despite mutation acquisition in hematopoietic stem cells, JMML-propagating cells are not always restricted to this compartment

**DOI:** 10.1038/s41375-020-0720-5

**Published:** 2020-01-31

**Authors:** Aurélie Caye, Kevin Rouault-Pierre, Marion Strullu, Elodie Lainey, Ander Abarrategi, Odile Fenneteau, Chloé Arfeuille, Jennifer Osman, Bruno Cassinat, Sabrina Pereira, Fernando Anjos-Afonso, Erin Currie, Linda Ariza-McNaughton, Vincent Barlogis, Jean-Hugues Dalle, André Baruchel, Christine Chomienne, Hélène Cavé, Dominique Bonnet

**Affiliations:** 1 INSERM UMR_S1131, Institut de Recherche Saint-Louis, Université de Paris, Paris, France; 2 Département de Génétique, Hôpital Robert Debré, Assistance Publique des Hôpitaux de Paris (AP-HP), Paris, France; 3 Francis Crick Institute, London, UK; 4 Service d’Hématologie Biologique, Hôpital Robert Debré, Assistance Publique des Hôpitaux de Paris (AP-HP), Paris, France; 5 Service de Biologie Cellulaire, Hôpital Saint Louis, Assistance Publique des Hôpitaux de Paris (AP-HP), Paris, France; 6 Service d’Hématologie Pédiatrique, Hôpital de la Timone, Assistance Publique des Hôpitaux de Marseille (AP-HM), Marseille, France; 7 Service d’Hématologie pédiatrique, Hôpital Robert Debré, Assistance Publique des Hôpitaux de Paris (AP-HP), Paris, France; 8 Present Address: Barts Cancer Institute, Centre for Haemato-Oncology, Queen Mary University of London, London, UK

**Keywords:** Cancer stem cells, Myelodysplastic syndrome

**Correction to: Leukemia**

10.1038/s41375-019-0662-y

This article was originally published under NPG’s License to publish, but has now been made available under a CC BY 4.0 license. The PDF and HTML versions of the paper have been modified accordingly.

