# Supplementary material for: Despite mutation acquisition in hematopoietic stem cells, JMML-propagating cells are not always restricted to this compartment
Source: Leukemia. 2019 Nov 27;34(6):1658–68. doi: 10.1038/s41375-019-0662-y (PMC7266742; doi:10.1038/s41375-019-0662-y)
Supplement: Supplementary file 1 — Supplementary data [file 41375_2019_662_MOESM1_ESM.docx]

**Supplementary Materials and Methods:**

**Phenotype and Isolation of CD34^+^ cells, HSC, MPP, LMPP, CMP, GMP and MEP**

Frozen primary patient samples were thawed and selected using an Easysep Human CD34 positive selection kit and Easysep magnet (StemCell Technologies, Vancouver, Canada) according to the manufacturer's instructions. CD34^+^ cells were stained with the antibodies described in the table Reagents and resources below. Cells were sorted as followed: HSC CD34^+^CD38^-^CD45RA^-^CD90^+^, MPP CD34^+^CD38^-^CD45RA^-^CD90^-^CD49f^-^, LMPP CD34^+^CD38^-^CD45RA^+^, CMP CD34^+^CD38^+^CD45RA^-^CD135^+^, GMP CD34^+^CD38^+^CD45RA^+^CD135^+^ and MEP CD34^+^CD38^+^CD45RA^-^CD135^-^. DAPI was added to the cell suspension prior sorting to exclude dead cells. Cells were sorted on a BD Influx^TM^ cell sorter (San Jose, CA) operating in 6-way purity sort mode and collected into 1.5 ml microfuge tubes.

**Colony-forming Cell assay**

*In vitro* growth assays of myeloid progenitors were performed by plating BM and/or peripheral blood mononucleated cells. At day 0, 500 isolated CD34^+^ obtained from patients were plated in semi-solid methylcellulose with or without leukocyte-conditioned medium (cytokine medium, LCM, cat no H4434 StemCell Technologies Inc, Vancouver, Canada), as previously described^1^. At termination of the mice, 10^5^ isolated human CD45^+^cells were plated in methylcellulose (cat no H4434 StemCell Technologies Inc, Vancouver, Canada). Colonies (containing >50 cells) were scored on days 11 and 14.

**Immunofluorescence of mouse bones, lung and spleen**

Harvested bones were fixed overnight in 10% neutral buffered formalin and then decalcified with 17% EDTA (Osteosoft, Millipore) for 7 days. Following on, bones were processed, paraffin embedded and sectioned (5 μm) for histological studies. Hematoxylin/eosin was performed first to assess quality of the sections. For immunofluorescence (IF) studies heat antigen retrieval was performed in all cases. Primary unconjugated antibodies employed were specific for the following proteins: human CD45 (Dako, M0701), Endomucin (Santa Cruz, sc-65495). Secondary fluorescent antibodies were from Invitrogen. Images were obtained using Zeiss Axio Scan.Z1 slice scanner and with Zen blue edition software. Data from images was obtained using Fiji software equipped with both grid overlay and Cell Counter plugins.

**Genomic DNA extraction:**

Peripheral blood and/or BM aspirates were collected on EDTA at diagnosis. Non-hematopoietic tissues (fibroblasts) were derived from skin biopsy for each patient. Genomic DNA was extracted from mononuclear cells, sorted cell fractions and fibroblasts using a Qiagen Mini or Midi Kit (Qiagen Gmbh). RNA was extracted from mononuclear cells using the miRNeasy Mini or Micro Kit (Qiagen). Concentrations were measured using a NanoDrop® (Thermo Scientific). cDNA was obtained by reverse transcription of 1 µg RNA with random hexamers, using standard procedures.

**Targeted sequencing on isolated colonies**

Colonies obtained from the *in vitro* growth of myeloid progenitors were picked and resuspended in 10 μl of TE buffer. Isolated colonies were lysed with proteinase K (10 μg) in 50 μl lysis buffer (50 mM KCl, 10 mM Tris-HCl pH 8, 2.5 mM MgCl2, 0.45% NP40, 0.45% Tween 20). Sanger direct sequencing was performed as mentioned below. 12 to 64 colonies were successfully screened per patient.

**RNAseq**

Libraries were prepared with TruSeq® Stranded Total RNASample preparation kit (Illumina) according to supplier’s recommendations. Briefly the ribosomal RNA fraction was removed from 1μg of total RNA using the Ribo-Zero™ Gold Kit (Epicentre). Fragmentation was then achieved using divalent cations under elevated temperature to obtain approximately 300bp pieces. Double strand cDNA synthesis was performed using reverse transcriptase and random primers, Illumina adapters were ligated and cDNA library was PCR amplified for sequencing. Paired-end 75b sequencing was then carried out on a HiSeq4000 (Illumina). Quality of reads was assessed for each sample using FastQC (<http://www.bioinformatics.babraham.ac.uk/projects/fastqc/>).

A subset of 500,000 reads from each Fastq file was aligned to the reference human genome hg19/GRCh37 with tophat2 to determine insert sizes with Picard. Full Fastq files were aligned to the reference human genome hg19/GRCh37 with tophat2 (-p 24 -r 150 -g 2 --library-type fr-firststrand). We removed reads mapping to multiple locations. We quantified gene expression using the full Gencode v26lift37. We used HTSeq to obtain the number of reads associated to each gene in the Gencode v26lift37 database (restricted to protein-coding genes, antisense and lincRNAs). We used the Bioconductor DESeq package to import raw HTSeq counts for each sample into R statistical software and extract the count matrix. After normalizing for library size, we normalized the count matrix by the coding length of genes to compute FPKM scores (number of fragments per kilobase of exon model and millions of mapped reads). Bigwig visualization files were generated using the bam2wig python script. CD90 overexpression was defined as a FPKM value >0.1 in at least 2 sorted progenitor fractions).

**Genome-wide DNA array analysis**

Genomic DNAs were analyzed by single-nucleotide polymorphism (SNP) array technologies using the Genome-Wide GeneChipR Human SNP Array 6.0 (Affymetrix) (n=7) or by high-density array comparative genomic hybridization (CGH) and SNP technologies using the SurePrint G3 Cancer CGH+SNP Microarray Kit, 4x180K (Agilent Technologies) (n=8), according to the manufacturers’ recommendations.

Analyses were performed using CytoGenomicsR (Agilent Technologies) for array CGH data, Genomic Suite 6.5 software (Partek), and the Hidden Markov Model and Segmentation algorithms for the analysis of both copy-number variations (CNV) and loss of heterozygosity (LOH). The final abnormalities retained were validated by visual analysis, considering the size and Log2 ratios of the abnormalities with respect to the individual background noise of each array at each particular chromosomal location.

Polymorphic CNVs were excluded using the Database of the Genomic Variants track in the University of California, Santa Cruz Genome Browser in the Cartagenia Bench Lab CNV software (Cartagenia). The human genome assembly GRCh37/hg19 was used as a reference.

**Whole-exome sequencing (WES)**

Targeted enrichment and massive parallel sequencing were performed on paired genomic DNA from leukocytes and fibroblasts. Exome capture was carried out using the SureSelect Human All Exon V4+UTRs or V5 or V5+UTRs or SureSelect Clinical Research (Agilent Technologies, Santa Clara, CA, USA) according to manufacturer’s instruction and protocols. Paired-end 75 bases sequencing was performed on a HiSeq2000 or HiSeq4000 instrument (Illumina, San Diego, CA, USA). Image analysis and base calling were performed using the Real Time Analysis (RTA) pipeline v. 1.14 (Illumina) with default parameters. The alignment of paired-end reads to the reference human genome (UCSC GRCh37/hg19), variant calling and generation of Quality variants scores were carried out using the CASAVA v.1.8 pipeline (Illumina).

Variant annotation was achieved using an in-house pipeline by IntegraGen (Evry, France) or the Ensembl’s VEP (Variant Effect Predictor, release 87) program. Gene and transcript names, strand, position (intronic, 5’UTR, 3’UTR…) were reported for each variant. Nucleotide, codon and amino acid changes as well as functional class (synonymous, missense, nonsense, splice…) were reported for coding variants.

Annotation content was compiled from several sources: 1000 genomes project and dbSNP, and/or ExAC and frequencies from IntegraGen Exome Databases which comprises 200 reference exomes. Finally, an in-house post-processing pipeline by IntegraGen (Evry, France) matched paired germline and tumor files in order to filter out candidate somatic mutations that are more consistent with artifacts. A somatic score ranging from 1 to 30 (a score of 30 translating the highest confidence index) was calculated for each variant, taking into account the counts and the frequencies of mutated allele in both samples.

For WES performed on xenotransplanted JMML, variant analysis was performed by comparison with WES data obtained on a non-transplanted NSG mouse in order to get rid of murine variants due to the presence of residual mouse cells in the purified samples.

Known polymorphisms reported at a frequency >0.1% in at least one of the above-mentioned databases, low coverage (<10 reads on germline and/or tumoral samples) and low-quality variants (Q variant score from IntegraGen ≤ 30) were systematically excluded.

**High-throughput targeted sequencing**

The coding region of 67 genes (full list provided in Table S5) previously shown to be altered in JMML was targeted by next generation sequencing after Custom SureSelectQXT enrichment (Agilent, Santa Clara, CA, USA). Design of the capture panel was performed using the web-based design tool eArray (Agilent). The target regions for each gene consisted in all the exons identified in NCBI RefSeq+/− 50 intronic flanking bp.

Libraries were performed by RNA-driven DNA capture following the manufacturer’s instructions. Briefly, 50 ng of genomic DNA was fragmented and adaptors were added in a single enzymatic step. The adaptor-tagged size-selected libraries were incubated with SureSelectQXT baits. RNA bait-DNA hybrids were then recovered using streptavidin-labeled magnetics beads. After RNA baits digestion, a post-capture PCR amplification was performed and samples were loaded for paired-end sequencing onto a NextSeq500R (Illumina, San Diego, CA, USA) using a Standard Flow Cell and a High Output Kit v2, 2*150bp.

Image analysis, alignment of sequence reads to the reference human genome build GRCh37 (hg19) and variant calling were performed on the BaseSpaceR (Illumina) using the BWA (Burrows-Wheeler Aligner) enrichment v2.1 pipeline (Illumina). To detect variants with variant allele frequency (VAF) <10%, variant calling was performed on a Galaxy instance (https://galaxy-public.curie.fr/) using VarScan v2.3.5 tool. When paired germline DNA was available, somatic variants were filtered using VarScan Somatic v2.3.5 and VarScan Process Somatic v1.0 tools, and CNV and aUPD were evaluated using Facets v1.0 tool.

Variant annotation and subsequent mutational analysis were performed according to international guidelines ^2^ using Bench Lab NGS, Cartagenia v5.0.1.

**Variant selection and classification**

Only somatically acquired variants (absent in germline sample but acquired in tumoral DNA or heterozygous in germline sample but homozygous in tumoral sample) with a probable impact at the protein level (non-synonymous exonic variants and abnormalities located at intron/exon junctions) and a variant allele frequency (VAF) higher or equal to 1% were considered for further analysis.

Previous involvement of confirmed somatic variants in cancer was verified by consulting the Catalogue for Somatic Mutations in Cancer (COSMIC). The prediction of functional effects of amino acid substitutions on the function and structure of proteins was achieved using dedicated prediction software: Scale-invariant feature transform (SIFT), Mutation Taster and PolyPhen-2.

**Sanger sequencing**

PCR was performed using the GoTaqR DNA Polymerase Kit (Promega) or the FastStartR Taq DNA Polymerase Kit (Roche) according to the manufacturer’s instructions. All primer sequences are listed in Table S8. PCR products were purified using the Illustra™ ExoStarR 1-Step kit (GE Healthcare, Life Sciences, Fairfield, USA) and direct sequencing was performed using the BigDye Terminator Ready Reaction Cycle Sequencing Kit (ABI, Foster City, CA, USA). Reaction products were run on an automated capillary sequencer (ABI 3130 Genetic Analyzer, ABI). Sequences were aligned using SeqscapeR analysis software (ABI) or visualized on ChromasR software (Technelysium) and compared with the reference sequences for genomic DNA.

**Supplementary References:**

1. Perez B, Kosmider O, Cassinat B, et al. Genetic typing of CBL, ASXL1, RUNX1, TET2 and JAK2 in juvenile myelomonocytic leukaemia reveals a genetic profile distinct from chronic myelomonocytic leukaemia. *Br J Haematol.* 2010;151(5):460-468.
2. Li MM, Datto M, Duncavage EJ, et al. Standards and Guidelines for the Interpretation and Reporting of Sequence Variants in Cancer: A Joint Consensus Recommendation of the Association for Molecular Pathology, American Society of Clinical Oncology, and College of American Pathologists. *J Mol Diagn.* 2017;19(1):4-23.

**Supplementary Tables & Figures legends:**

**Supplementary Figure S1**: **(A)** Gating strategy used for immune-phenotyping and cell sorting. **(B)** Distribution of cell fractions within the CD34^+^CD38^-^ (left panel) and the CD34^+^CD38^+^ populations (right panel), in BM from healthy patients and JMML according to the genetic group. NRAS- and KRAS-JMML were grouped for analysis (N/KRAS-JMML). Anova multiple comparison, ****: p< 0.0001; *< 0.05; ns: not significant. **(C)** Percentage of myeloid precursors within the CD34^+^/CD38^+^ compartment, comparing healthy aged matched children BM versus JMML samples according to the nature of initiating mutations. In *PTPN11*-JMML, myeloid precursors account for 73+/-12% (mean +/-SD) CD34^+^/38^+^ cells, vs 52+/-10% in the BM of healthy children (Anova multiple comparison: **: p< 0.01). ns: not significant.

**Supplementary Figure S2: Characterization of the *in vivo* model.**

**(A)** Kaplan-Meier representation of the percentage of healthy NSG or NSG-S injected with 7 and 6 JMML patient samples respectively. Mice were sacrificed when 20% reduction in body weight was reached. Statistical analysis was performed applying a Mantel-Cox test (Log-rank test), p value = 0.0016. **(B)** Comparison of 6-week engraftment of JMML (n=4) in NSG and NSG-S mice. **(C)** Kinetics of engraftment in NSG (at 6 weeks and 12 weeks) of 4 *PTPN11-JMML* and 1 *NRAS-JMML).* **(D)** May-Giemsa staining of bone marrow smears at diagnosis or post- xenotransplantation (x1000 magnification) (Left panel). Counts of blasts, neutrophils, monocytes and other cells at diagnosis and post-xenotransplantation for patients 88 (*PTPN11-JMML*), 92 (*NRAS*-JMML) and 66 (*KRAS-JMML*) (right panel). **(E)** Spleen size at sacrifice in NSG and NSG-S. NE: Non-engrafted, engrafted with patient 66 JMML (2 NSG & 2 NSG-S) and engrafted with patient 92 JMML (2 NSG & 2 NSG-S). **(F)** Table showing the respiratory distress observed in patients or the lung infiltration observed in xenotransplanted mice. In patient 53, infiltration of the mouse lung was present, although respiratory symptoms were not recorded in this patient. Right panel: Immunofluorescence pictures of the mice’s lung injected with samples from patient 50, 53, 66 and 92. In blue: Dapi; in red: endomucin; in green: human CD45.

**Supplementary Figure S3: Detailed analysis presented for four patients with multiple variants tested side by side in NSG and NSG-S models.**

Bar charts displaying frequencies of mutated alleles obtained by WES from samples at diagnosis, from NSG and from NSG-S engrafted samples retrieved at sacrifice into primary and secondary recipients. Among all variants studied, only the two *NF1* mutations, in patient 92, both displaying a VAF below 10% in native JMML, remained undetected in either NSG or NSG-S mice. Since these 2 variants were present in the same minor subclone, their absence in mice could be due to a stochastic process, the low number of injected cells (15000 CD34^+^ cells), reaching the limit of frequency of this subclone.

**Supplementary Figure S4**: **Clonal architecture of two JMML samples.**

Clonal architecture of 2 PTPN11-JMML samples: (#95) linear simple evolution, or (#88) showing independent acquisition of additional mutations targeting the RAS pathway (*PTPN11, NF1, CBL*) and JAK3. In the absence of preleukemia sample allowing to specify the kinetics of clonal emergence, subclones were represented by default as appearing simultaneously”.

See experimental schematic in Figure 4A. The clonal composition in total MNCs at diagnosis is represented in left side of the panel. Clonal composition and engraftment capacities across hematopoietic differentiation are represented on the right panels. Mutations identified in MNC were screened in sorted fractions before and after xenotransplantation using Sanger sequencing. Mouse icons tag fractions that were injected in NSG and/or NSG-S mice. Red mouse icons indicate successful engraftment whereas grey icons indicate engraftment failure, with an asterix in case the failure is likely to be due to insufficient cells injected.

**Supplementary table S1**

**Supplementary Table S1:** Details of the different data generated by sample of the patients’ cohort (n=36). Thirteen patients were previously included in the study by Caye *et al.,* ^5^ using the same ID. GL: germline.

**Supplementary table S2**

**Supplementary Table S2: Phenotype analysis of healthy versus JMML BM samples**

Heat map representation (with percentage) of phenotypic data in JMML. The different HSPCs populations’ distribution in patients with JMML of different genetic subtypes, and healthy children bone marrow. HSC, MPP and LMPP are compared to each other within the CD34^+^CD38^-^ fraction, and CMP, GMP & MEP are compared to each other within the CD34^+^CD38^+^ fraction. The distribution of CD90/Thy1 expression is expressed as the percentage of CD90/Thy1 expressing positive cells according to the fraction of origin in patients with JMML of different genetic subtypes, and healthy children bone marrow.

**Supplementary Table S3**: Secondary transplantation experiment. Experimental information and percentage of engraftment of the serially transplanted samples in NSG versus NSG-S mice. (see also Figure 3C).

**Supplementary Figure S4**

**Supplementary Table S4**: Mutational landscape and variant allele frequencies of the JMML that were xenotransplanted.

**Supplementary Table S5**

**Supplementary Table S5**: Variant allele frequency at diagnosis, relapse, and post-xenotransplantation.NA: not applicable.

**Supplementary Table S6**

**Supplementary Table S6**: Analysis of the sequencing of colonies and cells out of mice in comparison to the VAF (%) obtained at diagnosis/relapse for patients 88, 66 and 92. The first column “clones” represents the different clones observed in patient samples along with the mutations they harbored as revealed by the colony assay. The second column “comments” highlights the clonal hierarchy inferred from the sequencing data, xenotransplantation results and colonies analysis. The columns “VAF” display the different VAF observed at diagnosis/relapse or after xenotransplantation. The VAF of the initiating clone (IC) was evaluated applying the following equation: VAF_IC_ = VAF_initiating mutation_ – (Sum of VAF_clones a,b,c…_). In the different CFC columns, the fraction in brackets shows the number of positive colonies / total colonies, and in the same box this fraction has been translated into percentage (%). VAF that were inferred are indicated *in italics*. NA: not available. *aUPD: acquired uniparental disomy.

**Supplementary Table S7**

**Supplementary Table S7**: Mutation landscape across the isolated stem and progenitor hematopoietic fractions for the different patients. VAF% is indicated in case samples tested by NGS. NA: not applicable. mut: mutation present.

**Supplementary table S8:** Reagents and resources used in this paper.

| REAGENT or RESOURCE | SOURCE | IDENTIFIER |
| --- | --- | --- |
| Antibodies | | |
| Dapi | BD Biosciences | 564907 |
| CD2 APC | BD Biosciences | 341024 |
| CD19 FITC | BD Biosciences | 555412 |
| CD33 PE | BD Biosciences | 555450 |
| CD34 PerCP-Cy5.5 | BD Biosciences | 347203 |
| CD34 APC-Cy7 | Biolegend | BLE343514 |
| CD38 PE-Cy7 | eBioscience | 25-0388-42 |
| CD38 FITC | Biolegend | BLE303504 |
| Human CD45 PE-Cy7 | eBioscience | 25-0459-42 |
| Mouse CD45 PerCP-Cy5.5 | BD Biosciences | [550994](https://www.bdbiosciences.com/us/applications/research/stem-cell-research/cancer-research/mouse/percp-cy55-rat-anti-mouse-cd45-30-f11/p/550994) |
| CD45RA AF780 | eBioscience | 47-0458-42 |
| CD45RA PerCP-Cy5.5 | Biolegend | BLE304122 |
| Mouse CD45 FiTc | eBioscience | 11-0451-85 |
| Human CD45 PE | BD Biosciences | 555483 |
| CD49f PE | BD Biosciences | 555736 |
| CD64 PE | DAKO | R7219 |
| CD90 PE-Cy7 | Biolegend | BLE328124 |
| CD90 APC | eBioscience | 17-0909-42 |
| CD135 BV711 | BD Biosciences | 563908 |
| CD135 AF647 | BD Pharmingen | 563494 |
| Human CD45 | DAKO | M070129-2 |
| Endomucin | Santa Cruz | Sc-65495 |
| Secondary Ab AF647 Goat anti-rat | ThermoFisher | A-21247 |
| Secondary Ab AF594 Goat anti-mouse | ThermoFisher | A-11005 |
| Secondary Ab AF488 Goat anti-mouse | ThermoFisher | A-11029 |
| Biological Samples | | |
| JMML samples | Robert Debré hospital biobank |  |
| Children BM | Robert Debré hospital biobank |  |
| Deposited Data | | |
| Whole exome sequencing | This paper | ArrayExpress database: <http://www.ebi.ac.uk/arrayexpress>  Accession number E-MTAB-6461 (patients) and E-MTAB-6467 (xenograft samples) |
| SNP/CGH array | This paper | ArrayExpress database: <http://www.ebi.ac.uk/arrayexpress>  Accession numbers E-MTAB-3729 (SNP aray) and E-MTAB-6468 (CGH+SNP array) |
| Experimental Models: Organisms/Strains | | |
| NOD/SCID/IL2rγ−/− (NSG) mice | The Jackson Lab / breed at the Francis Crick Institute | 005557 |
| NOD/SCID/IL2rγ−/−/IL-3/GM/SF (NSG-S) mice | The Jackson Lab / breed at the Francis Crick Institute | 013062 |
| Oligonucleotides | | |
| Primers for PCR and Sanger sequencing | This paper | Table S4 & S5 (see below) |
| Software and Algorithms | | |
| FlowJoTM 10 | FlowJo LLC |  |
| Prism 7.0c | GraphPad |  |
| Facets 1.0 | Shen R. el al, [Nucleic Acids Res.](https://www.ncbi.nlm.nih.gov/pubmed/27270079) 2016 |  |
| VarScan 2.3.5 |  | varscan.sourceforge.net/somatic-calling.html |
| Other | | |
| Genome Browser | University of California, Santa Cruz (UCSC) | http://www.genome.ucsc.edu/ |
| dbSNP137 | NIH | <http://www.ncbi.nlm.nih.gov/projects/SNP> |
| HapMap | NIH | <http://hapmap.ncbi.nlm.nih.gov/> |
| 1,000 Genomes |  | <http://www.1000genomes.org/> |
| Catalogue of Somatic Mutations in Cancer (COSMIC), |  | http://cancer.sanger.ac.uk/cosmic |
| SIFT |  | <http://sift.jcvi.org/> |
| PolyPhen-2 |  | <http://genetics.bwh.harvard.edu/pph2/> |
| MutationTaster |  | <http://www.mutationtaster.org/> |
| Leiden Open Variation Database (LOVD) | Leiden university | http://www.lovd.nl/3.0/ |
